# Indoor air pollution concentrations and cardiometabolic health across four diverse settings in Peru: a cross-sectional study

**DOI:** 10.1186/s12940-020-00612-y

**Published:** 2020-06-03

**Authors:** Josiah L. Kephart, Magdalena Fandiño-Del-Rio, Kirsten Koehler, Antonio Bernabe-Ortiz, J. Jaime Miranda, Robert H. Gilman, William Checkley

**Affiliations:** 1 Department of Environmental Health and Engineering, Bloomberg School of Public Health, Johns Hopkins University, Baltimore, MD USA; 2 Center for Global Non-Communicable Disease Research and Training, Johns Hopkins University, Baltimore, MD USA; 3 Present Address: Urban Health Collaborative, Dornsife School of Public Health, Drexel University, Philadelphia, PA USA; 4 CRONICAS Center of Excellence in Chronic Diseases, Universidad Peruana Cayetano Heredia, Lima, Peru; 5 School of Medicine, Universidad Peruana Cayetano Heredia, Lima, Peru; 6 Program in Global Disease Epidemiology and Control, Department of International Health, Bloomberg School of Public Health, Johns Hopkins University, Baltimore, MD USA; 7 Division of Pulmonary and Critical Care, School of Medicine, Johns Hopkins University, 1830 E. Monument St Room 555, Baltimore, MD 21287 USA

**Keywords:** Indoor air pollution, Particulate matter, Carbon monoxide, Blood pressure, Exhaled carbon monoxide, C-reactive protein, Haemoglobin A1c, Air pollution epidemiology, Peru, Latin America

## Abstract

**Background:**

Indoor air pollution is an important risk factor for health in low- and middle-income countries.

**Methods:**

We measured indoor fine particulate matter (PM_2.5_) and carbon monoxide (CO) concentrations in 617 houses across four settings with varying urbanisation, altitude, and biomass cookstove use in Peru, between 2010 and 2016. We assessed the associations between indoor pollutant concentrations and blood pressure (BP), exhaled carbon monoxide (eCO), C-reactive protein (CRP), and haemoglobin A1c (HbA1c) using multivariable linear regression among all participants and stratifying by use of biomass cookstoves.

**Results:**

We found high concentrations of indoor PM_2.5_ across all four settings (geometric mean ± geometric standard deviation of PM_2.5_ daily average in μg/m^3^): Lima 41.1 ± 1.3, Tumbes 35.8 ± 1.4, urban Puno 14.1 ± 1.7, and rural Puno 58.8 ± 3.1. High indoor CO concentrations were common in rural households (geometric mean ± geometric standard deviation of CO daily average in ppm): rural Puno 4.9 ± 4.3. Higher indoor PM_2.5_ was associated with having a higher systolic BP (1.51 mmHg per interquartile range (IQR) increase, 95% CI 0.16 to 2.86), a higher diastolic BP (1.39 mmHg higher DBP per IQR increase, 95% CI 0.52 to 2.25), and a higher eCO (2.05 ppm higher per IQR increase, 95% CI 0.52 to 3.57). When stratifying by biomass cookstove use, our results were consistent with effect measure modification in the association between PM_2.5_ and eCO: among biomass users eCO was 0.20 ppm higher per IQR increase in PM_2.5_ (95% CI − 2.05 to 2.46), and among non-biomass users eCO was 5.00 ppm higher per IQR increase in PM_2.5_ (95% CI 1.58 to 8.41). We did not find associations between indoor air concentrations and CRP or HbA1c outcomes.

**Conclusions:**

Excessive indoor concentrations of PM_2.5_ are widespread in homes across varying levels of urbanisation, altitude, and biomass cookstove use in Peru and are associated with worse BP and higher eCO.

## Introduction

Air pollution is a growing threat to public health in low- and middle-income countries (LMICs) [1] and is estimated to be responsible for 4.9 million deaths globally in 2017 [2]. Air pollution can be broadly divided into ambient air pollution and indoor air pollution (IAP). Ambient air pollution can be produced by mobile sources such as vehicular exhaust, point sources such as power plants, natural processes such as windborne dust, or IAP which has escaped outdoors. IAP within a house is typically a mixture of ambient air pollution that has infiltrated the house and pollution produced within the house by household activities such as cooking, cleaning, or smoking [3].

Historically, public health research has focused on ambient air pollution [4], using measurements from fixed monitoring sites to estimate outdoor concentrations at an individual’s residence, which is often used a proxy for personal exposure. However, many people in LMICs spend a majority of their time indoors. In a study in rural Mexico, adult women spent 76% of time indoors [5], while people in urban areas generally spend even more time indoors than rural populations [6]. Ambient and indoor pollutant concentrations often have inconsistent correlation and in many settings indoor concentrations are higher than ambient concentrations [7]. Exposure-response relationships which rely on estimates of ambient pollutant concentrations [8] are vulnerable to misclassification of true pollutant exposures in populations who spend a majority of time indoors [6].

A major source of indoor air pollution in LMICs is the use of biomass fuels such as dung, wood, agricultural residue, or charcoal for cooking and heating. Three billion people globally use biomass cookstoves [9] and exposure to the resulting air pollution is a leading risk factor for the global burden of disease, responsible for 1.6 million premature deaths in 2017 [2]. Pollution from biomass cookstoves, known as household air pollution (HAP), is often characterized by high-dose concentration spikes and substantial variability throughout the day associated with cooking events [7, 10, 11]. Compared to homes with biomass cookstoves, indoor concentrations can be relatively consistent in homes where IAP is primarily driven by ambient pollution infiltrating the house [10]. Such variations in the temporal patterns of exposures may have distinct health effects but are poorly captured with time-weighted average (TWA) sampling methods [7], which are unable to capture concentration spikes by design.

The indoor air pollutants of greatest public health concern include fine particulate matter (PM_2.5_) and carbon monoxide (CO) [12]. Epidemiological studies of ambient air pollution have found associations between PM_2.5_ concentrations and a higher risk of cardiovascular-related morbidity and mortality [13–16]. There is growing evidence of associations between ambient PM_2.5_ exposure and other cardiometabolic outcomes, including blood pressure [17–19] and diabetes [20]. Inflammation is thought to play a major role in the impact of PM_2.5_ on cardiometabolic health [13], and intermediary markers of inflammation, such as C-reactive protein and exhaled carbon monoxide (eCO), have been associated with both long-term and short-term particulate matter exposure [21–24]. Chronic CO exposures have also been linked to congestive heart failure, ischemic heart disease, and cardiovascular disease [25], as well as increased C-reactive protein [26]. While there is growing evidence for relationships between ambient air pollution and cardiometabolic health, there are few studies [27, 28] that explore the impact of IAP on cardiometabolic health in LMICs and very few studies that examine IAP in LMIC homes that use gas, electricity, or other non-biomass fuels.

This study aims to characterize indoor concentrations of PM_2.5_ and CO in homes across four settings in Peru, which are diverse in urbanisation, altitude, and use of biomass fuels, and to examine the cross-sectional associations between single- and multi-pollutant IAP concentrations and cardiometabolic outcomes. We hypothesize that IAP concentrations will vary widely by setting and that these concentrations will be associated with negative effects on cardiometabolic health. We also aim to assess these concentration-response relationships independently among biomass cookstove users and non-biomass cookstove users to examine the possibility of biomass use as an effect measure modifier of these concentration-response relationships. We hypothesize that negative associations between IAP concentrations and health outcomes will vary by use of biomass cookstoves.

## Methods

### Study design and setting

CRONICAS is a longitudinal cohort study that seeks to explore prevalence and trends in chronic diseases across four sites in Peru with varying altitude and urbanicity [29]. The study enrolled participants from the following sites: Pampas de San Juan de Miraflores, a peri-urban community with 50,000 inhabitants located approximately 25 km south of central Lima at sea level; Tumbes, a group of communities with approximately 20,000 people on the northern coast of Peru at sea level that is comprised of a mix of agriculture and rapidly developing urban areas; Puno city, an urban area of approximately 230,000 inhabitants located at 3825 m above sea level on the shores of Lake Titicaca; and rural Puno, a region of low-density agricultural communities surrounding Puno city, where use of biomass fuels is prevalent. This study received ethical approval from Institutional Review Boards at Universidad Peruana Cayetano Heredia, Asociación Benéfica PRISMA, and the Johns Hopkins Bloomberg School of Public Health. Additional information on the CRONICAS cohort study has been previously published [29].

### Study population and sampling

Participants were sampled by a sex- and age-stratified random sample from a local census performed by study staff in each source community. The minimum age for inclusion was 35 years of age, as the study was designed to examine the incidence and progression of chronic diseases, which are more common at later ages. Exclusion criteria included women who were pregnant, individuals who were unable to give consent, and anyone with a physical disability that would prevent measurements of blood pressure or anthropometrics. While eligible for enrolment in the CRONICAS cohort study, in this analysis we excluded participants who reported taking antihypertensive medications from the blood pressure analysis, participants who reported receiving diabetes treatment from the HbA1c analysis, and participants who reported smoking cigarettes daily from the eCO and CRP analyses. A maximum of one participant per household was considered for inclusion in the study. Questionnaires were collected at baseline, 15 months post-baseline, and 30 months post-baseline between 2010 and 2014. Participants were asked to report age, sex, current medical diagnoses and treatments, sociodemographic information, daily use of a biomass cookstove (yes/no), frequency of alcohol consumption, and salt consumption (five categorical responses ranging from “a lot” to “a little”). We created a wealth index based on the assets (iron, colour TV, computer, cell phone, etc.) and facilities (piped water, material of roof, floor, etc.) available in the household of each participant. A weighted sum of the assets and facilities was calculated for each participants’ household and the resulting index was divided in tertiles [30]. Blood pressure was measured at baseline, 15 months post-baseline, and 30-months post-baseline. Venous blood samples were collected at baseline and 30-months post-baseline. IAP was sampled for 48 h once per household during the follow-up period. Clinical measurements were collected between 2010 and 2014, and IAP assessments were conducted between 2013 and 2016.

### Indoor air pollution assessment

Particulate matter was sampled by nephelometric methods using the DataRAM pDR-1000 (Thermo Fisher Scientific, Waltham, MA, USA), which has a concentration measurement range of 0.001–400 mg/m^3^ and a resolution of the larger of 0.001 mg/m^3^ or 0.1%. None of the measured concentrations were above 400 mg/m^3^ and measurements below 0.001 mg/m^3^ were replaced with 0.0005 mg/m^3^. Relative humidity (RH) was recorded with HOBO RH data loggers (Onset Computer Corporation, Bourne, MA, USA). Real-time PM concentrations were RH-adjusted and converted to gravimetric-equivalent PM_2.5_ concentrations using a global gravimetric-correction equation. This correction was developed previously for the DataRAM pDR-1000 by concurrently sampling nephelometric and gravimetric concentrations over 24 h in 32 urban and 72 rural homes in Peru with a mix of biomass and non-biomass fuel types [10]. CO was assessed using the EL-USB-CO data logger (Lascar Electronics, Erie, PA, USA), with a measurement range of 0 to 1000 ppm and a resolution of 1 ppm. No measured concentrations exceeded 1000 ppm and readings below 1 ppm were replaced with 0.5 ppm. PM, RH, and CO monitors were co-located in the kitchen area for 48 h and measurements were recorded every minute. Samples that did not reach a minimum of 24 h were excluded from the analysis.

### Clinical assessment

Systolic and diastolic blood pressure were taken using a HEM-780 automatic monitor (Omron Healthcare, Inc., Mississauga, Ontario, Canada). Blood pressure measurements were taken in triplicate and the final two measurements were averaged to obtain the final values. Participant standing height was measured in triplicate using standardized methods and weight was measured using the TBF-300A body composition analyser (TANITA Corporation, Tokyo, Japan). eCO was assessed using the Micro CO meter (Micro Direct, Lewiston, ME, USA) and monitors were calibrated monthly. A trained technician collected 13.5 ml of venous blood after 8–12 h of participant fasting. Highly sensitive C-reactive protein was assessed using Latex (Tina-quant CRP-HS Roche/Hitachi analyser, Indianapolis, IN, USA) and haemoglobin A1C (HbA1c) was analysed using high performance liquid chromatography (D10, BioRad, Munich, Germany). Detailed information on the clinical assessment, blood sampling, and laboratory analysis has been previously published [29].

### Biostatistical methods

The primary analytical aims of this analysis were to characterize indoor concentrations of PM_2.5_ and CO across four diverse settings in Peru and to examine the cross-sectional concentration-response associations between indoor PM_2.5_ and CO and summary measures of systolic and diastolic blood pressure, eCO, CRP, and HbA1c. A secondary analysis aimed to stratify participants by use of biomass cookstoves and determine if concentration-response relationships vary by biomass use. Additionally, in the concentration-response analyses of blood pressure, we stratified by sex and age (< 50 years vs. > − 50 years) and examined associations independently.

PM_2.5_ and CO measurements across multiple calendar days were averaged by calendar minute to create equally time-weighted daily mean concentrations. We also calculated the proportion of daily time spent over the 24-h WHO indoor air quality guidelines for PM_2.5_ (25 μg/m^3^) [25, 31] and CO (7 mg/m^3^ or ~ 5.68 ppm) [25] to characterize the duration of excessive indoor concentrations within the day. As air quality measurements and clinical outcomes were often assessed at different times and the goal of the study was to capture long-term clinical status, we used the average of all available longitudinal clinical measurements for each outcome from each participant. This included a total of three BP measurements (baseline, 15 months, 30 months), one eCO measurement at 30 months, and two CRP and HbA1c measurements (baseline and 30 months).

We used multivariable linear regression to evaluate associations between PM_2.5_ and/or CO concentrations and clinical outcomes. Associations for each clinical outcome were examined in both single and multi-pollutant (PM_2.5_ and CO) models. For each outcome, we limited the analysis to complete cases to allow for directly comparability between single- and multi-pollutant models. All regression models were adjusted for age, sex, body mass index (BMI), wealth index tertile (lowest tertile as reference), and living at high altitude (both rural and urban Puno are in a high-altitude plateau approximately 3825 m above sea level, whereas both Lima and Tumbes are coastal cities at approximately sea level). We included BMI, wealth, and high altitude as complementary correlates of potentially confounding factors relating to lifestyle (modern to traditional), physiological differences related to altitude, as well as regional social differences between Andean and coastal populations. We also examined alcohol consumption (no alcohol or any alcohol in the past month) and self-reported salt consumption as potential confounders, and we included alcohol or salt consumption in the final multivariable models when associated with the health outcome at a significance level of *p* ≤ 0.10. IAP concentrations were log-transformed for the concentration-response analysis based on a WHO precedent of using a log-linear concentration-response curve to estimate cardiopulmonary morbidity [32] and to comply with linear model assumptions of homoscedasticity. To assist interpretability, we scaled the association between clinical outcomes and IAP concentrations to an increase in the interquartile range (IQR) of observed PM_2.5_ or CO concentrations (e.g.,

1 $$ \hat{\beta}\times \log \frac{75^{th} percentile\ in\ {PM}_{2.5}}{25^{th} percentile\ in\ {PM}_{2.5}} $$

We refer to the difference between the 75th and 25th percentiles in observed IAP concentrations as an IQR increase.

We excluded from the blood pressure analysis 85 participants who reported taking blood pressure medications. We similarly excluded 20 participants who reported receiving diabetes treatment from the HbA1c analysis. Additionally, 49 and 12 participants who reported smoking cigarettes daily were excluded from the eCO and CRP analyses, respectively. Following the primary analysis with all participants, participants were stratified by self-reported daily use of a biomass cookstove and analysed independently using the same multivariable linear models described above. All analyses were performed using R (www.r-project.org) [33].

## Results

A total of 617 households were successfully sampled for PM_2.5_, CO, and clinical measurements **(**Table 1**)**. The number of participants from each setting varied from 92 participants in Tumbes to 254 participants in rural Puno. Participants were broadly similar across settings by sex (overall 53.8% female) and age (overall mean 57.1 years), while other clinical and behavioural variables varied by setting. For example, in Tumbes, alcohol use was lowest, yet cigarette use was highest when compared to other settings. Daily use of biomass cookstoves was reported by 46.2% of all participants and varied by site, from very low use in Lima (5.7%) and urban Puno (5.4%) to moderate use in Tumbes (27.2%) and near-universal use in rural Puno (96.5%).

**Table 1:** Demographic, clinical, behavioral, and environmental characteristics of 617 participants from four diverse settings in Peru

	**All**		**Lima**	**Tumbes**	**Puno City**	**Puno Rural**	**All biomass users**	**All non-biomass users**
	Total N	N (%) or Mean (SD)	N (%) or Mean (SD)	N (%) or Mean (SD)	N (%) or Mean (SD)	N (%) or Mean (SD)	N (%) or Mean (SD)	N (%) or Mean (SD)
Number of Participants	617		105 (17.0%)	92 (14.9%)	166 (26.9%)	254 (41.2%)	285 (46.2%)	332 (53.8%)
Female	617	332 (53.8%)	62 (59.0%)	47 (51.1%)	89 (53.6%)	134 (52.8%)	158 (55.4%)	174 (52.4%)
Age in years	589	57.1 (12.4)	57.2 (10.3)	57.9 (13.2)	56.5 (12.4)	57.3 (13.0)	58.1 (13.0)	56.3 (11.9)
Wealth index tertile	617							
1 (lowest)		262 (42.5%)	13 (12.4%)	22 (23.9%)	43 (25.9%)	184 (72.4%)	195 (68.4%)	67 (20.2%)
2		186 (30.1%)	41 (39.0%)	43 (46.7%)	39 (23.4%)	63 (24.8%)	76 (26.7%)	110 (33.1%)
3 (highest)		169 (27.4%)	51 (48.6%)	27 (29.3%)	84 (50.6%)	7 (2.8%)	14 (4.9%)	155 (46.7%)
Body Mass Index (BMI)	572	27.3 (4.5)	28.6 (3.9)	29.4 (5.3)	28.1 (4.1)	25.5 (4.0)	25.9 (4.2)	28.5 (4.3)
Obese (BMI ≥ 30)	572	129 (22.6%)	32 (31.4%)	33 (38.8%)	38 (25.0%)	26 (11.2%)	36 (13.7%)	93 (30.1%)
Systolic Blood Pressure (mmHg)	613	115 (16)	117 (16)	125 (18)	112 (16)	111 (13)	113 (14)	116 (17)
Diastolic Blood Pressure (mmHg)	613	73 (9)	71 (9)	76 (9)	71 (9)	73 (8)	73 (8)	72 (9)
Blood pressure treatment	613	85 (13.9%)	24 (22.9%)	27 (29.3%)	20 (12.3%)	14 (5.5%)	23 (8.1%)	62 (18.8%)
Previous hypertension diagnosis	617	95 (15.4%)	24 (22.9%)	23 (25.0%)	29 (17.5%)	19 (7.4%)	28 (9.8%)	67 (20.2%)
Exhaled carbon monoxide (ppm)	587	11.8 (12.8)	3.4 (2.0)	17.2 (12.7)	9.3 (11.0)	15.2 (14.3)	15.1 (14.2)	9.0 (10.6)
C-reactive protein (mg/L)	589	2.9 (5.9)	3.9 (5.0)	5.3 (9.7)	2.7 (3.3)	1.9 (5.4)	2.1 (5.3)	3.7 (6.2)
Hemoglobin A1c %	589	5.9 (1.0)	5.9 (1.2)	5.9 (0.7)	6.0 (1.4)	5.7 (0.5)	5.8 (0.6)	6.0 (1.2)
Previous diabetes diagnosis	617	19 (3.1%)	3 (2.8%)	5 (5.4%)	8 (4.8%)	3 (1.2%)	4 (1.4%)	15 (4.5%)
Alcohol in past year	613	348 (56.8%)	78 (74.3%)	24 (26.1%)	86 (52.7%)	160 (63.2%)	162 (57.2%)	186 (56.3%)
Daily cigarette smoking	585	12 (2.1%)	3 (2.9%)	7 (8.2%)	2 (1.2%)	0 (0.0%)	1 (0.4%)	11 (3.5%)
Daily use of biomass cookstove	617	285 (46.2%)	6 (5.7%)	25 (27.2%)	9 (5.4%)	245 (96.5%)	285 (100%)	332 (100%)

**Fig. 1:**
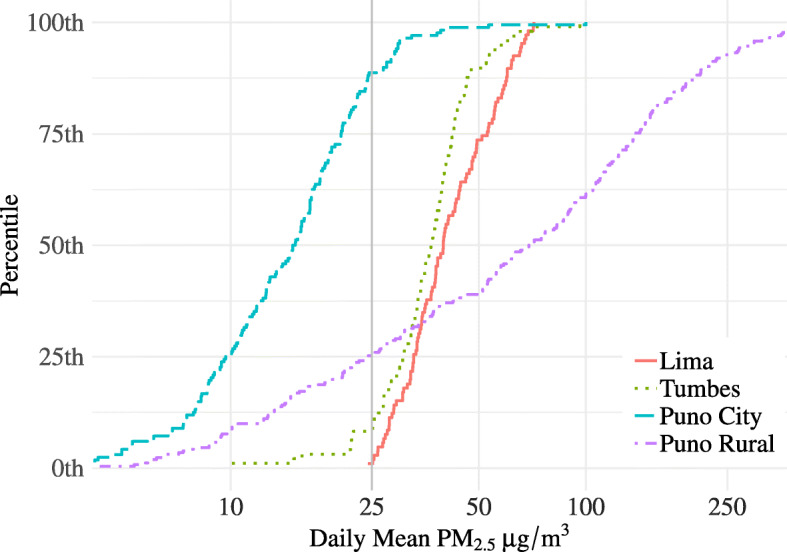
Distributions of daily mean indoor PM_2.5_ concentrations in 617 houses across four sites in Peru

PM_2.5_ samples were completed in 617 households. The geometric mean (GM) daily average indoor PM_2.5_ concentration was 41.1 μg/m^3^ (geometric standard deviation [GSD] 1.3) in Lima, 35.8 μg/m^3^ (GSD 1.4) in Tumbes, 14.1 μg/m^3^ (GSD 1.7) in urban Puno, and 58.8 μg/m^3^ (GSD 3.1) in rural Puno **(**Table 2**)**. Nearly all homes in Lima and Tumbes had daily mean PM_2.5_ concentrations exceeding WHO 24-h guidelines for PM_2.5_ (25 μg/m^3^) [31], while approximately 75% of houses in rural Puno and 10% of houses in urban Puno exceeded the same guidelines. An empirical cumulative distribution plot of daily mean concentrations by site (Fig. 1) demonstrates relatively narrow variability in mean concentrations in Lima, Tumbes, and urban Puno, indicating similar indoor concentrations within these settings despite differences in absolute concentrations across settings. In contrast, there is wide variability in rural Puno, where biomass cookstoves are prevalent, and observed indoor concentrations span three orders of magnitude.

**Table 2:** Distribution of indoor air pollution concentrations in 617 houses in Peru, by site and use of biomass cookstoves

	**Lima**	**Tumbes**	**Puno City**	**Puno Rural**	**All Biomass Users**	**All Non-biomass Users**
	N or Mean (SD)	N or Mean (SD)	N or Mean (SD)	N or Mean (SD)	N or Mean (SD)	N or Mean (SD)
Number of households	105	92	166	254		
**PM**_**2.5**_**μg/m**^**3**^**24-h means**						
Mean (SD)	42.8 (12.4)	37.6 (12.1)	16.3 (10.3)	99.6 (102.0)	92.1 (98.1)	29.2 (20.5)
Geometric mean (GSD)	41.1 (1.3)	35.8 (1.4)	14.1 (1.7)	58.8 (3.1)	55.0 (2.9)	23.7 (2.0)
Daily hours > 25 μg/m^3^	20.7 (4.3)	18.3 (6.2)	3.6 (3.1)	5.0 (3.1)	6.5 (5.8)	11.6 (9.1)
Spearman correlation: Daily mean vs. hrs. > 25 μg/m^3^	0.87	0.82	0.91	0.59	0.47	0.95
**CO ppm 24-h means**						
Mean (SD)	1.3 (0.9)	0.9 (0.6)	1.9 (3.2)	12.1 (17.9)	10.8 (17.2)	1.5 (2.4)
Geometric mean (GSD)	1.0 (2.0)	0.8 (1.6)	1.2 (2.3)	4.9 (4.3)	4.0 (4.4)	1.0 (2.1)
Daily hours > 5.68 ppm	0.2 (0.5)	0.3 (0.9)	1.3 (2.6)	6.0 (6.7)	5.4 (6.6)	0.8 (1.9)
Spearman correlation: Daily mean vs. hrs. > 5.68 ppm	0.51	0.49	0.91	0.92	0.93	0.73

**Fig. 2:**
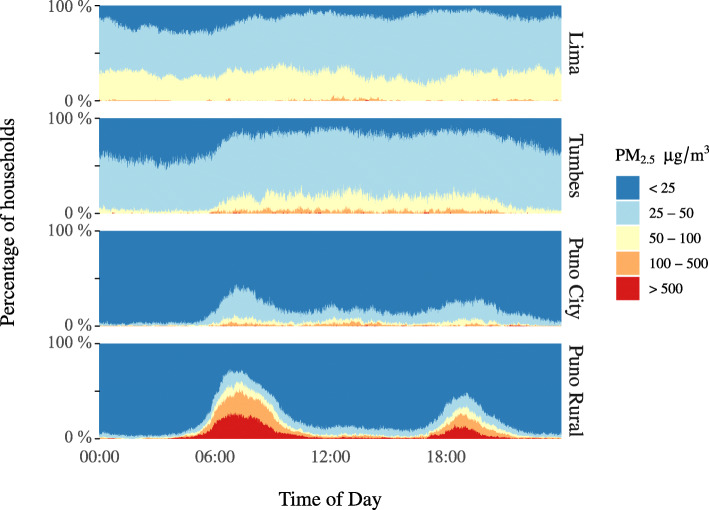
Indoor PM_2.5_ concentrations by calendar minute in 617 houses across four sites in Peru

By examining the data as a bar plot of the distribution of concentrations within each site at each minute of the calendar day (Fig. 2), we observe important differences in temporal patterns. This figure displays the proportion of households which fall into a given concentration category at any given time of day, stratified by study site. In Lima and Tumbes, concentrations were relatively consistent, generally between 25 and 100 μg/m^3^, without substantial variation by time of day. In Puno city, concentrations were similarly consistent throughout the day but remained generally below the WHO 24-h air quality guidelines (dark blue colour), with some increases in the prevalence of moderate concentrations during waking hours. In contrast, rural Puno demonstrates low concentrations at night and high concentrations between 5 A.M. and 9 A.M. with a second, smaller spike between 5 P.M. and 8 P.M., times in which many people are preparing food at the start and the end of the workday.

**Fig. 3:**
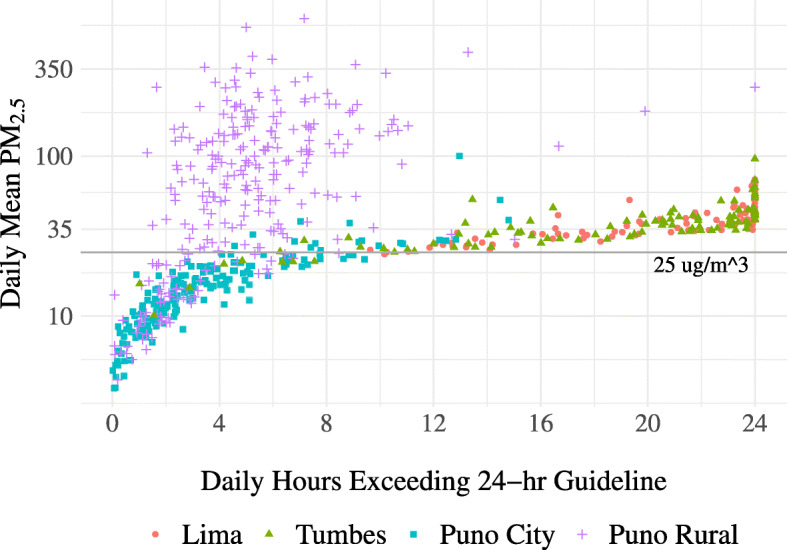
Daily mean indoor PM_2.5_ concentrations and daily hours spent in excess of WHO annual guidelines

Differences in concentration profiles across sites were also represented by differences in the correlation between mean concentrations and the duration of time with concentrations in exceedance of 24-h WHO air quality guidelines (Fig. 3**)**. In Lima and Tumbes, mean concentrations were high overall and were above guidelines for a large proportion of the day. Puno city had a similar correlation between mean concentrations and duration of excessive concentration, but with lower concentrations and less time spent above guidelines (Table 2**)**. In contrast, mean concentrations in rural Puno were excessively high, but these concentrations were distributed over relatively few hours of the day as short-duration, high concentration spikes.

**Fig. 4:**
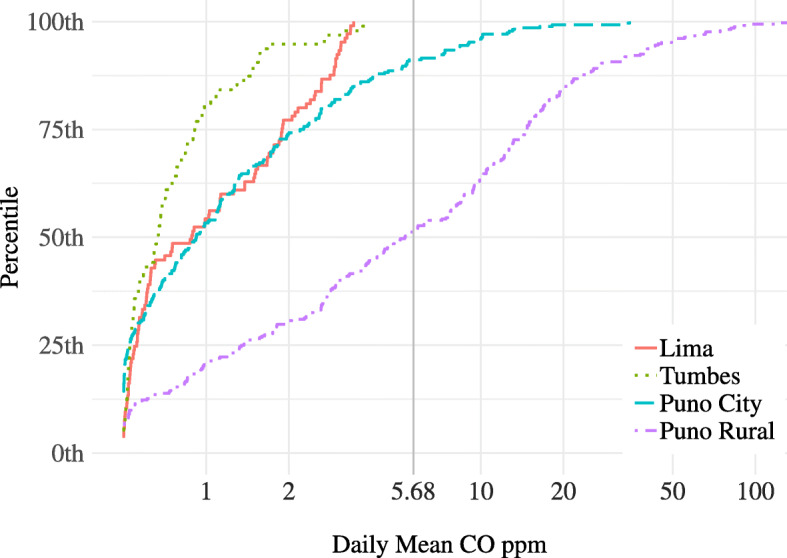
Daily mean indoor CO concentrations in 617 houses across four diverse sites in Peru

We also collected indoor CO samples for 617 households. The GM daily average indoor CO concentration was 1.0 ppm (GSD 2.0) in Lima, 0.8 ppm (GSD 1.6) in Tumbes, 1.2 ppm (GSD 2.3) in Puno city, and 4.9 ppm (GSD 4.3) in rural Puno (Table 2). All kitchens sampled in Lima and Tumbes had daily mean concentrations below the WHO 24-h guidelines [25]. Similar to PM_2.5_, we observed wide variability in concentrations within households in rural Puno (Fig. 4).

**Fig. 5:**
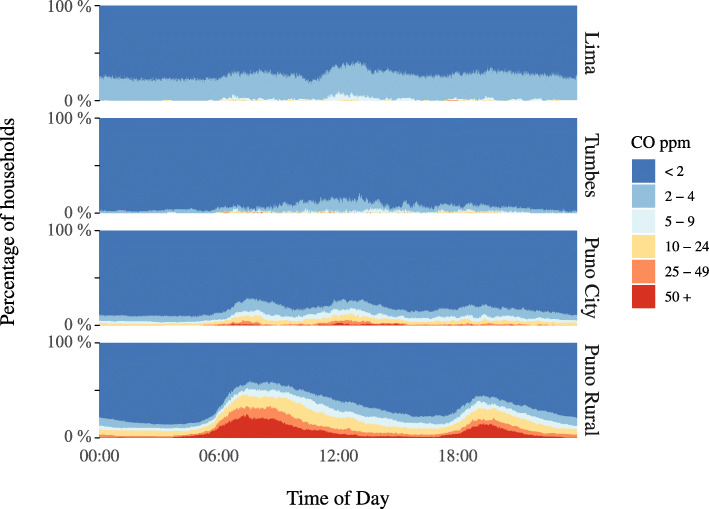
Indoor CO concentrations by calendar minute in 617 houses across four diverse sites in Peru

In Lima and Tumbes, the distribution of CO concentrations was consistent throughout the day (Fig. 5), while in urban Puno, approximately 10% of households had excessive concentrations during waking hours. In rural Puno, a pattern similar to PM_2.5_ was apparent, with higher concentrations around the morning and evening cooking times.

Correlation between daily mean PM_2.5_ and CO concentrations were overall low but varied by site. Generally, the three sites with lower biomass cookstove use had low Spearman correlations of mean PM_2.5_ and mean CO (Lima − 0.08, Tumbes 0.15, urban Puno 0.14). In rural Puno, where biomass use is near-exclusive, PM_2.5_ and CO were moderately correlated (Spearman correlation 0.66). Overall, the Spearman correlation between mean PM_2.5_ and CO concentrations among biomass-using households was 0.68, and the correlation among households that did not use biomass fuels was − 0.05.

In the adjusted, single-pollutant model for systolic blood pressure (SBP), an IQR increase in PM_2.5_ (25th percentile 17 μg/m^3^, 75th percentile 60 μg/m^3^) was associated with a higher SBP of 1.51 mmHg (95% CI 0.16 to 2.86) (Table 3). An IQR increase in CO (25th percentile 0.6 ppm, 75th percentile 4.9 ppm) in the multi-pollutant model was associated with a slightly lower SBP of − 0.17 mmHg (95% CI − 2.38 to 2.03). For diastolic blood pressure (DBP), an IQR increase in PM_2.5_ in the adjusted, multi-pollutant model was associated with a higher DBP of 1.42 mmHg (95% CI 0.28 to 2.56), whereas an IQR increase in CO was not associated with DBP (− 0.06 mmHg, 95% CI − 1.48 to 1.35).

**Table 3:** Multivariable linear regression of indoor air pollutants and associated differences in cardiometabolic outcomes

	**All Participants**	**Biomass Users**	**Not Biomass Users**
**Model**	**Estimate (95% CI)**	**Estimate (95% CI)**	**Estimate (95% CI)**
**Systolic Blood Pressure (mmHg)**^**a**^			
Number of observations	488	237	251
PM_2.5_	1.51 (0.16, 2.86)^*^	1.49 (−0.14, 3.12)	1.08 (−2.85, 5.02)
CO	1.12 (− 0.55, 2.79)	1.50 (− 0.52, 3.52)	−1.72 (−5.70, 2.26)
Multipollutant: PM_2.5_	1.60 (− 0.18, 3.39)	1.22 (− 0.96, 3.40)	1.55 (− 2.49, 5.59)
Multipollutant: CO	−0.17 (− 2.38, 2.03)	0.50 (− 2.21, 3.20)	−2.07 (−6.16, 2.01)
**Diastolic Blood Pressure (mmHg)**^**a**^			
Number of observations	488	237	251
PM_2.5_	1.39 (0.52, 2.25)^*^	0.86 (− 0.18, 1.91)	0.37 (− 2.13, 2.86)
CO	1.08 (0.01, 2.16)^*^	0.91 (− 0.39, 2.20)	−1.59 (−4.11, 0.92)
Multipollutant: PM_2.5_	1.42 (0.28, 2.56)^*^	0.67 (− 0.73, 2.07)	0.76 (− 1.80, 3.32)
Multipollutant: CO	−0.06 (− 1.48, 1.35)	0.35 (−1.38, 2.09)	− 1.77 (− 4.36, 0.82)
**Exhaled Carbon Monoxide (ppm)**^**b**^			
Number of observations	519	247	272
PM_2.5_	2.05 (0.52, 3.57)^*^	0.20 (−2.05, 2.46)	5.00 (1.58, 8.41)^*^
CO	1.75 (− 0.10, 3.60)	1.02 (− 1.24, 3.29)	−1.51 (−3.94, 0.91)
Multipollutant: PM_2.5_	1.90 (− 0.09, 3.89)	−0.93 (− 3.90, 2.05)	5.30 (1.81, 8.79)^*^
Multipollutant: CO	0.28 (−2.12, 2.68)	1.49 (− 2.07, 5.04)	−1.32 (− 4.72, 2.09)
**C-reactive protein (mg/L)**^**b**^			
Number of observations	556	260	296
PM_2.5_	−0.58 (− 1.24, 0.09)	−0.22 (− 0.98, 0.55)	−2.03 (− 3.96, − 0.10)^*^
CO	−0.25 (− 1.06, 0.57)	−0.10 (− 1.04, 0.84)	−0.13 (− 2.06, 1.79)
Multipollutant: PM_2.5_	−0.76 (− 1.64, 0.11)	−0.29 (− 1.32, 0.75)	− 2.08 (− 4.05, − 0.11)^*^
Multipollutant: CO	0.36 (−0.71, 1.42)	0.13 (−1.13, 1.39)	0.26 (− 1.70, 2.21)
**Hemoglobin A1c %**^**b**^			
Number of observations	549	260	289
PM_2.5_	− 0.05 (− 0.11, 0.01)	−0.05 (− 0.13, 0.02)	0.02 (− 0.15, 0.20)
CO	−0.05 (− 0.13, 0.02)	−0.08 (− 0.17, 0.02)	0.05 (− 0.12, 0.23)
Multipollutant: PM_2.5_	−0.03 (− 0.12, 0.05)	−0.02 (− 0.12, 0.08)	0.01 (− 0.17, 0.20)
Multipollutant: CO	−0.03 (− 0.13, 0.08)	−0.06 (− 0.19, 0.07)	0.05 (− 0.13, 0.23)

^a^Adjusted for age, sex, body mass index (BMI), alcohol consumption, high altitude, and household wealth

^b^Adjusted for age, sex, BMI, high altitude, and household wealth

^*^p-value < 0.05

Compared to all participants, we observed slightly weaker concentration-response associations between PM_2.5_ and both SBP and DBP when stratifying biomass and non-biomass users, with no evidence of biomass use as an effect measure modifier. In the adjusted, multi-pollutant model, an IQR increase in PM_2.5_ was associated with a 1.22 mmHg (95% CI − 0.96 to 3.40) higher SBP among biomass users, and a 1.55 mmHg (95% CI − 2.49 to 5.59) higher SBP among non-biomass users. We found no evidence of an association between indoor CO concentrations and BP among biomass users or non-users. Additional analyses found no evidence of a difference in concentration-response associations between pollutants and BP when stratifying by sex or by age (< 50 years vs. ≥ 50 years, results not presented).

In the adjusted, single-pollutant model, an IQR increase in PM_2.5_ was associated with a higher eCO (2.05 ppm, 95% CI 0.52 to 3.57) (Table 3). After stratifying by biomass use, the effect size was nearly doubled in non-biomass users (5.00 ppm, 95% CI 1.58 to 8.41) yet was attenuated among biomass users (0.20 ppm, 95% CI − 2.05 to 2.46), which is consistent with biomass use as an effect measure modifier. This strong association among non-biomass users persisted in the multi-pollutant analysis (IQR increase in PM_2.5_ associated with 5.30 ppm [95% CI 1.81 to 8.79] higher eCO). We found no evidence of an concentration-response association between CO and eCO.

We found no statistically significant associations between indoor PM_2.5_ or CO concentrations and CRP in the single or multipollutant models (Table 3). In the adjusted, multipollutant model, IQR increases in PM_2.5_ and CO were associated with differences in CRP of − 0.76 mg/L (95% CI − 1.64 to 0.11) and 0.36 mg/L (95% CI − 0.71 to 1.42), respectively. When stratifying by biomass use, we observed an unexpected negative association between PM_2.5_ and CRP among non-biomass users and no association among biomass users.

We found no significant associations between indoor concentrations of PM_2.5_ or CO and HbA1c **(**Table 3**)**. In the adjusted, multi-pollutant model, an IQR increase in PM_2.5_ was associated with a higher HbA1c of − 0.03% (95% CI − 0.12 to 0.05). Similarly, an IQR increase in CO was associated with a higher HbA1c of − 0.03% (95% CI − 0.13 to 0.08).

## Discussion

This study used a population-based random sample of adults to characterize indoor air pollution concentrations and cardiometabolic outcomes in four settings in Peru. In Peru and in LMICs more broadly, the vast majority of previous exposure assessments of indoor air pollution have been limited to rural households which use a specific cooking fuel of interest, such as a wood burning fire [34] or exclusive use of biomass [35]. While these exposure estimates are useful for evaluating cookstove-related exposures, a population-based sample, as used in the current study, provides a better estimate of the burden of indoor pollution borne by a population as a whole. Additionally, most previous exposure assessments in urban areas of LMICs assign individuals exposure estimates derived from ambient air pollution models. By taking direct measurements of IAP at participant homes, where many people spend a majority of their time, we use concentration estimates which may be more relevant to true exposure than modelled ambient concentrations. Lastly, this study provides, to our knowledge, the first direct measurements of indoor residential CO in a population-based sample in coastal Peru, where 50% of the national population lives.

In this population-based study of adults in Peru, we found widespread indoor concentrations of PM_2.5_ which exceed WHO indoor guidelines across four diverse settings in Peru with wide-varying urbanisation and use of biomass cookstoves. CO concentrations were entirely within WHO indoor 24-h guidelines in the urban areas of Lima and Tumbes, yet approximately 50% of households in rural Puno had daily mean concentrations that exceeded these guidelines. By using direct-reading air quality monitors at one-minute temporal resolution, we were able to observe large differences between sites in the temporal profiles of pollutant concentrations throughout the day. In Lima and Tumbes, PM_2.5_ concentrations were similar between houses and stable throughout the day, suggesting that in these urban, coastal settings individual household behaviours may have a limited role in determining indoor concentrations compared to external factors, such as ambient air pollution. In contrast, IAP concentrations in rural Puno were widely varying between households, with dramatic spikes during common mealtimes, suggesting the dominance of household behaviours and biomass cookstoves as a source of IAP. We also found evidence of a positive association between indoor PM_2.5_ concentration and higher blood pressure among a diverse group of individuals in urban and rural Peru representing both ambient- and biomass cookstove-dominated sources of indoor pollution. We found a positive association between indoor PM_2.5_ and eCO and evidence that suggests biomass use as a potential effect measure modifier of this concentration-response relationship.

The indoor PM_2.5_ concentrations which we observed in rural Puno (daily mean 99.6 μg/m^3^) were similar in range than previous assessments in the Puno region. Pollard et al. [10] previously observed a median of mean 24-h concentrations of 130 μg/m^3^ in rural Puno. At the same site, we observed similar CO concentrations (median of the means 5.3 ppm) than previous literature (Pollard, median of means 5.8 ppm [10]). In Lima, we found indoor PM_2.5_ concentrations (mean 42.8 μg/m^3^) somewhat higher than previous indoor assessments in the same city by Underhill et al. [36] (mean 20 μg/m^3^) and Robinson et al. [37] (median 31 μg/m^3^). In Tumbes, concentrations of indoor PM_2.5_ were higher in this study (median 32.4 μg/m^3^) than a previous assessment by Robinson et al. [37] (median 13 μg/m^3^), although the assessment by Robinson et al. did not include gravimetric correction. We are unaware of prior assessments of indoor, residential CO in urban, coastal Peru. PM_2.5_ and CO daily means were moderately correlated in biomass-using houses and not correlated in non-biomass using houses. This supports a recent systematic review [38] that describes the limitations of using CO as a correlate for PM_2.5_ in household air pollution exposure assessments.

We observed a positive association between IAP with BP which is consistent with findings from previous studies looking at cookstove-related IAP in Guatemala [35], Bolivia [39], Honduras [28], and China [27]. This association has also been noted with ambient PM_2.5_ [17–19]. We observed a 1.51 mmHg higher SBP per IQR increase in PM_2.5_, suggesting that participants within the highest quantiles of observed indoor PM_2.5_ concentration have clinically meaningful differences in systolic BP (≥ 2 mmHg difference) [40] compared to participants with lower observed indoor PM_2.5_ concentrations. While previous studies have found a stronger association between biomass-related PM_2.5_ and BP among older women than in younger women [27, 28], we did not find evidence to support this finding, We also did not find a difference in the association between PM_2.5_ and BP stratified by sex (results not presented). The observed association between PM_2.5_ and eCO is consistent with previous findings that eCO can be a useful biomarker of exposure to smoke from a variety of sources [10, 22, 24]. We found no association between IAP and CRP or HbA1c, and the current literature is inconclusive. Given our sample sizes for CRP (*n* = 556) and HbA1c (*n* = 549), our adjusted, single-pollutant models could have detected a 3% increase in R^2^ with 0.89 and 0.89 power, respectively. However, this ancillary study was not designed to measure an association between blood biomarkers and environmental exposures, and there is in many cases a temporal mismatch between the timing and duration of the indoor air pollution measurements and the ideal exposure windows represented by the biomarkers. Specifically, CRP has a plasma half-life of 19 h [41], and we did not assess IAP concentration on the previous or same day of blood collection. Additionally, our 48-h assessments of IAP may not represent an individual’s chronic exposures which we would expect to be more related to HbA1c, a measure of long-term diabetes progression. This analysis makes the assumption that both the biomarker samples and 48-h IAP assessments represent long-term outcomes and exposures, respectively. The HbA1c levels in this study (mean of all participants 5.87%) were similar to a previous assessment of HbA1c levels in Peruvian populations at sea-level (5.9%) and high-altitude (5.8%) [42].

We found the association between PM_2.5_ and eCO to be strengthened in non-biomass users and attenuated in biomass users after stratification by cookstove type. This corroborates a study in 2015 by Caravedo et al. in the Puno region which found lower CRP among biomass users when compared to non-biomass users [43]. These differences could be a result of different temporal exposure profiles, with indoor concentrations among biomass users frequently characterized by short duration, high dose concentration spikes while indoor concentrations among non-biomass users are more commonly stable and chronic. These unique temporal profiles may have distinct impacts on health, which are inadequately captured by using long-term mean concentrations to pool concentration-response estimates from different sources (e.g. ambient PM_2.5_ and cookstove-related PM_2.5_). The association between CO and eCO was attenuated in the multipollutant model compared to the single pollutant model among all participants and among non-biomass users. This could suggest that exposure to PM_2.5_ is a stronger predictor of eCO through inflammation-related pathways, or that CO exposures which influence eCO levels, such as ambient or occupational exposures to CO, were not captured by residential CO measurements.

This study has various strengths, including its use of individual-level clinical and environmental data from a random, population-based sample. We used consistent environmental and clinical assessment methods to observe participants in four diverse settings in a middle-income country, from major urban centres to rural agricultural areas. Additionally, we evaluated the concentration-response relationships with directly measured indoor air pollutant concentrations at the household level in place of concentration estimates derived from modelling ambient air pollution. This study was limited by clinical and environmental data that were typically not sampled on the same day and in some cases environmental measurements were collected after clinical data. Because of this limitation, we averaged all available clinical measurements throughout time for each participant to better capture long-term outcome status throughout the study period. Furthermore, indoor air quality was assessed on one occasion for 24–48 h. This snapshot of IAP concentration, combined with the temporal mismatch between IAP and clinical assessments requires the assumptions that IAP concentrations captured during the environmental assessment meaningfully represent long-term concentration patterns within a given household and that the longitudinal clinical data similarly represents chronic disease status. Additionally, while using household pollutant concentrations to characterize an individual’s exposure has benefits over ambient estimates, personal exposure assessments are the ideal method to accurately classify an individual’s true exposure. Our analysis used BMI, a wealth index, and high altitude (a measure of Andean vs coastal region) as proxies for region within Peru and for lifestyle factors which are potential confounders for the associations between IAP and clinical outcomes. While in full models which included BMI and altitude we found no evidence of salt consumption having a substantial impact on any of the examined associations, and alcohol consumption only have an association with blood pressure outcomes, it is likely that BMI, wealth, and altitude are insufficient to fully adjust for all relevant and unmeasured confounders. We also did not collect information on ambient air pollution or on specific cookstove and kitchen characteristics which may explain variations in IAP concentrations within and across the four settings. Unobserved kitchen characteristics may also relate to true personal exposures to IAP, such as proximity and ventilation between the kitchen, the rest of the house, and the outdoors. We were unable to collect information on time spent indoors or individual participants’ involvement with cooking activities.

## Conclusions

A large proportion of households across four Peruvian settings with varying urbanisation, altitudes, and household behaviours have indoor PM_2.5_ concentrations which exceed WHO indoor guidelines. In rural Puno, where biomass use is prevalent, excessive concentrations of CO are also common. In urban homes without biomass cookstoves IAP concentrations are generally stable throughout the day, while in homes with biomass cookstoves, regardless of urbanisation, IAP concentrations are characterized by short duration, high concentration spikes. We found evidence to support the association of indoor PM_2.5_ and SBP, DBP, and eCO. The concentration-response relationship between PM_2.5_ and eCO varied between biomass users and non-biomass users, with a stronger positive relationship among non-biomass users which could be explained by dramatically different temporal concentration profiles. Further research is warranted to explore this phenomenon.

## Data Availability

The datasets used and/or analyzed during the current study are available from the corresponding author on reasonable request.

## Abbreviations

BMI: Body mass index
BP: Blood pressure
CO: Carbon monoxide
CRP: C-reactive protein
eCO: Exhaled carbon monoxide
GM: Geometric mean
GSD: Geometric standard deviation
HAP: Household air pollution
HbA1c: Haemoglobin A1c
IAP: Indoor air pollution
IQR: Interquartile range
LMIC: Low- and middle-income countries
PM2.5: Fine particulate matter
RH: Relative humidity
TWA: Time-weighted average
